# Complex-Value Coherence Mapping Reveals Novel Abnormal Resting-State Functional Connectivity Networks in Task-Specific Focal Hand Dystonia

**DOI:** 10.3389/fneur.2013.00149

**Published:** 2013-10-10

**Authors:** Leighton B. N. Hinkley, Kensuke Sekihara, Julia P. Owen, Kelly P. Westlake, Nancy N. Byl, Srikantan S. Nagarajan

**Affiliations:** ^1^Department of Radiology and Biomedical Imaging, University of California San Francisco, San Francisco, CA, USA; ^2^Department of Systems Design and Engineering, Tokyo Metropolitan University, Tokyo, Japan; ^3^Department of Physical Therapy and Rehabilitation Science, University of California San Francisco, San Francisco, CA, USA

**Keywords:** sensorimotor, coherence, functional connectivity, resting state, dystonia

## Abstract

Resting-state imaging designs are powerful in modeling functional networks in movement disorders because they eliminate task performance related confounds. However, the most common metric for quantifying functional connectivity, i.e., bivariate magnitude coherence (Coh), can sometimes be contaminated by spurious correlations in blood-oxygen level dependent (BOLD) signal due to smoothing and seed blur, thereby limiting the identification of true interactions between neighboring neural populations. Here, we apply a novel functional connectivity metric., i.e., imaginary coherence (ICoh), to BOLD fMRI data in healthy individuals and patients with task-specific focal hand dystonia (tspFHD), in addition to the traditional magnitude Coh metric. We reconstructed resting-state sensorimotor, basal ganglia, and default-mode networks using both Coh and ICoh. We demonstrate that indeed the ICoh metric eliminates spatial blur around seed placement and reflects slightly different networks from Coh. We then identified significant reductions in resting-state connectivity within both the sensorimotor and basal ganglia networks in patients with tspFHD, primarily in the hemisphere contralateral to the affected hand. Collectively, these findings direct our attention to the fact that multiple networks are decoupled in tspFHD that can be unraveled by different functional connectivity metrics, and that this aberrant communication contributes to clinical deficits in the disorder.

## Introduction

Task-specific focal hand dystonia (tspFHD) is a movement disorder characterized by involuntary end range twisting postures of the fingers, wrist, and forearm that only arise during the performance of a well-rehearsed motor behavior (e.g., writing, piano playing). Current working hypotheses of tspFHD propose that it is a disorder of functional connectivity that develops when aberrant receptive fields in primary sensorimotor regions introduce gain downstream into motor association areas [e.g., pre-motor cortex, supplementary motor area (SMA)] and the basal ganglia producing aberrant co-contractions of the digits and hand (1). These hypotheses are supported by findings from non-invasive neuroimaging studies in tspFHD, which have demonstrated heightened activity in both the basal ganglia and cortical regions of the sensorimotor network (SMN) [see Ref. (2–4) for a review].

Abnormal neural activity in tspFHD has been extensively studied using both PET (5) and fMRI (6–9) and these studies have shown that both primary (e.g., M1, S1) and association regions of motor cortex (e.g., SMA) as well as basal ganglia regions (e.g., putamen, caudate, globus pallidus) exhibit increased activity during task performance in patients when compared to controls. EEG and MEG imaging studies of tspFHD have also provided evidence that oscillatory activity and coupling is compromised in these patients over sensorimotor cortices (10–13). Collectively, these imaging studies suggest that abnormal activation in the cortex and basal ganglia during behavior are inherent to tspFHD.

In cognitive and translational neuroscience, one technique that has gained popularity in recent years is to examine correlations in the blood-oxygen level dependent (BOLD) signal derived from functional MRI recordings in the absence of behavior, an approach known as resting-state functional connectivity (14, 15). Resting-state imaging designs permit the examination of spontaneous activity and connectivity in the brain. Since the participant is not engaged in any task in resting-state designs, they permit the identification of functionally specialized neural networks that are not confounded by differences in performance or cognitive load. It has been argued that resting-state functional imaging is especially powerful for examining patient populations, where subject compliance and ability to perform a task during scanning can become an issue (16, 17).

Quantification of resting-state functional connectivity is most commonly done using bivariate metrics of interactions between the BOLD signal timeseries of two separate elements (voxels or regions) measured during fMRI recording. Here, a “seed” (either a single point or cluster of voxels centered on a region of interest) is placed in the volume and functional connectivity is estimated between the seed region and the rest of the brain. The most dominant of these bivariate metrics are temporal correlation and magnitude coherence (Coh), and they have been effectively applied to identify networks during behavior serving sensory perception (18), motor control (19, 20), and cognition (21). However, magnitude Coh and correlations exhibit a phenomena known as “seed blur,” where commonalities in HRF morphology and between neighboring voxels and processing artifacts such as spatial smoothing produce spuriously high correlations between two voxel timeseries. As a result, it may be difficult to discern regions around a seed voxel that show high functional connectivity that are due to this spatial blur from regions representing true neurophysiologically coupled brain interactions. Imaginary coherence (ICoh) is a metric sensitive only to non-zero time-lagged coupling between two source elements, and is therefore argued as a true brain measure of neural synchronization (22). Since many fMRI functional connectivity studies demonstrate significant seed blur, due to potentially spurious correlations between neighboring voxels, it is possible that the examination of time-lagged functional interactions in these datasets permits the visualization of interactions free of artifacts such as seed blur. It is also possible that functional connectivity metrics like ICoh will elucidate complementary cortical and sub-cortical networks not typically seen using metrics like correlation and Coh.

The goal of the present study is to further delineate functional abnormalities within the sensorimotor and basal ganglia networks (BGNs) in tspFHD using resting-state fMRI using both standard (magnitude Coh) and novel (ICoh) metrics of functional connectivity. First, we characterize both instantaneous and time-lagged BOLD resting-state networks in a sample of healthy controls (HCs). We propose that many of the limitations inherent to fMRI functional connectivity studies (e.g., truncated synchronous networks, seed blur) are due to a dominance of zero-lag networks derived from correlation/Coh metrics, and that ICoh will reduce or even eliminate many of these confounds. Second, we will examine differences in the SMN, BGN, and default-mode network (DMN) in patients with tspFHD. We predict that alterations in neural activity identified in neuroimaging studies contribute to a disruption of cooperation (and a reduction of connections) between functionally relevant neural networks in tspFHD. We further predict that the sensitivity of ICoh to BOLD fMRI networks with non-zero time lag will permit us to examine many of the motor system interactions inaccessible to classical functional connectivity measures.

## Materials and Methods

### Participants

All procedures were approved by the UCSF Committee on Human Research, and all experiments were conducted in accordance with the Declaration of Helsinki. All participants gave written informed consent following explanation of study procedures. Eleven patients with tspFHD were recruited from the UCSF Faculty Practice in Physical Therapy and the Movement Disorders Clinic at UCSF. Of the 11 tspFHD patients (mean age 48, SD = 9 years), 10 were right handed (two left hand affected), and 1 was left handed (left hand affected). Seven of the patients were diagnosed with writer’s cramp, three with musician’s cramp, and one with occupational cramp. All patients with tspFHD were diagnosed by a neurologist specializing in movement disorders prior to enrollment. All patients were screened for any secondary dystonia and a subset of patients (*n* = 5) were clinically evaluated for severity using the Burke–Fahn–Mardsen dystonia movement scale (23) and ranged from moderate to severe. None of the patients included in this study were currently receiving botulinum toxin (Botox) or other treatments. History of Botox treatment was variable across our sample, however, those who were included in the study did not have any injections 3 months prior. All patients were diagnosed with tspFHD at least 1 year prior to enrollment in the study. In order to be included in this study, patients had to be between 21 and 75 years of age, possess clear dystonic movements related to the performance of a target task and have no specific neurological disorder that would explain the signs and symptoms. Exclusion criteria included: systemic or neurologic disease associated with a known movement disorder, medical instability, and electromagnetically activated medical equipment or devices which might cause damage to the sensitive detection circuits. Patients with systemic of neurologic disease associated with a known movement disorder, medical instability, Botox injections within the 3-months prior to participation in the study and contraindications for MRI were excluded from this study. In addition, 15 HC participants aged 29–75 years (mean age 56.9, SD = 14 years) were recruited from the greater San Francisco Bay Area for enrollment in this study. Of these 15 HC participants, 7 were female and 14 were right handed.

### MRI acquisition

MRI data was acquired using a 3.0-T Siemens Trio (Siemens, Erlangen, Germany) installed at the UCSF Neuroscience Imaging Center (NIC). For each subject, a high-resolution anatomical MRI was acquired (MPRAGE; 160 1 mm slices, FOV = 256 mm, TR = 2300 ms, TE = 2.98 ms). Eight minutes (240 repetitions) of spontaneous fMRI data was collected (supine position, eyes closed) with a gradient EPI sequence (38 3.0 mm × 3.0 mm × 3.0 mm slides, TR = 2000 ms, TE = 28 ms).

### Data processing

Resting-state fMRI data was spatially pre-processed using SPM5. All images were realigned and spatially normalized to the EPI template (resliced and interpolated into 3 mm isotropic voxels). Spatially normalized images were smoothed using a 10-mm FWHM kernel [as in Ref. (14)] and read into MATLAB for functional connectivity analysis. Data from all voxels was linearly detrended and bandpass filtered (second-order Butterworth; 0.01–0.08 Hz) prior to functional connectivity analysis.

### Seed definition

Seeds were placed in five regions of interests (ROIs) based on previous resting-state fMRI studies of the motor (14, 19, 20, 24) and default-mode (15) networks. ROI seeds consisted of a sphere with a 5-mm radius and defined using the MaRSBAR Matlab toolbox (http://marsbar.sourceforget.net) consisting of 16–23 3 mm voxels. For primary motor cortex (M1), anatomically defined seeds were centered on the “hand knob” region of the Rolandic sulcus of the left and right hemispheres. For the basal ganglia, a seed was placed in the putamen of the left and right hemispheres based on the center of mass of this region on the MNI template brain. The putamen was chosen for the BGN as the networks seeded in this region overlap functional connectivity maps seen when seeds are placed in other BG structures (caudate, globus pallidus) and is relevant to studies of tspFHD, as aberrations in this structure in patients have been reported in previous imaging studies (25). As dystonic symptoms are generally more severe (or affected) in a single hand in tspFHD, both M1 and putamen seed maps for the tspFHD group were split based on representations of the “affected” (i.e., contralateral to the most severely affected hand) and “unaffected” (i.e., contralateral to the least affected hand) hands [as in Ref. (26, 60)]. For the DMN, a single seed was placed over posterior cingulate cortex (PCC) based on coordinates provided from other resting-state fMRI studies (28).

### Functional connectivity analysis and group statistics

Details about the estimation of parameters for Coh and ICoh analyses are presented in detail elsewhere (19, 22, 29, 30). Briefly, Coh between elements *x* and *y* is defined as the absolute value of the normalized cross-spectrum (*S*) of coherency:

$${\text{Coh}}_{xy}\left(f\right)=\left|\frac{S_{xy}\left(f\right)}{{\left(S_{xx}\left(f\right)S_{yy}\left(f\right)\right)}^{1/2}}\right|$$

Although the Coh metric is intended to represent true interactions between voxels *x* and *y*, estimation of this interaction inherently includes signal leakage (*d*) between a target voxel and its neighboring voxels. This leakage can, in turn, contribute to spurious auto-correlations that arise from commonalities in the hemodynamic signal shared between the two voxels. Therefore, even when there is no true source interaction present, Coh can have non-zero value, caused by the autocorrelation between *x* and its leakage (*d_x_*) with *y* and its leakage (*d_y_*). However, when the imaginary portion of the Coh cross-spectrum is extracted from this estimate, non-zero values indicate that no spurious correlation has been generated [see Ref. (31)]. ICoh is defined as the regression between the residual and the seed voxel (i.e., anything outside of instantaneous correlations), therefore removing any artifacts generated from common sources (including seed blur). ICoh is defined as the absolute value of the imaginary component of coherency, which accounts for a portion of the Coh spectrum sensitive to interactions with a degree of time lag (22, 30):

$${\text{iCoh}}_{xy}\left(f\right)=\left|\text{lm}\frac{S_{xy}\left(f\right)}{{\left(S_{xx}\left(f\right)S_{yy}\left(f\right)\right)}^{1/2}}\right|$$

Both Coh and ICoh estimates were generated for each subject between each voxel in the seed and all remaining voxels in the reconstruction. Each voxel’s matrix of connectivity values (*C*) were Fisher’s *Z*-transformed in order to give a averaged global measure of connectivity for that location. In order to statistically normalize the data for group analysis, each individual subject’s functional connectivity map was transformed (coefficient of determination) prior to both group averaging and second-level statistics. In order to quantify differences in the magnitude of resting-state functional connectivity between HC and tspFHD groups, a subset of participants from the larger HC sample (*n* = 11) were selected and group matched on both handedness (10 right handed), gender (6 female), and age (mean = 52.36 years, unpaired *t*-test for age between HC and tspFHD groups, *p* = 0.17). Voxelwise comparisons between groups (HC, tspFHD) were performed using parametric unpaired *t*-tests. As three of the tspFHD patients were left hand affected, functional connectivity maps for these patients were flipped (transformed in the *x* plane along the mid sagittal axis) prior to group analyses so that, for all patients, the left hemisphere represented the “most affected” hemisphere [as in Ref. (32)].

## Results

### Default-mode network

**Figure 1:**
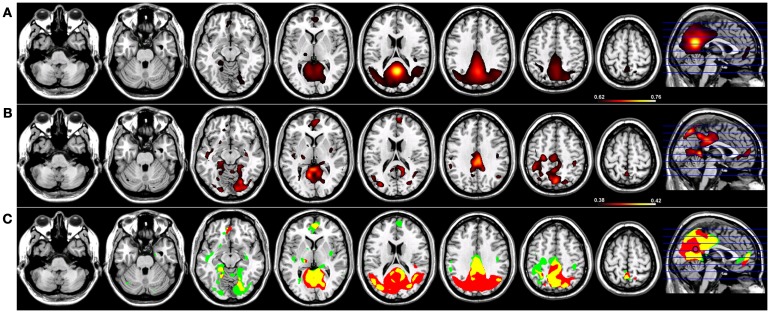
**Resting-state functional connectivity maps for a seed placed in posterior cingulate cortex (pink circle) in 15 healthy control subjects reconstructing the default-mode network (DMN)**. **(A)** DMN reconstruction using magnitude coherence (Coh). **(B)** DMN reconstruction using imaginary coherence (ICoh). **(C)** Spatial conjunction of Coh maps (in red) and ICoh maps (in green) for the DMN and overlap between the two maps (in yellow). All maps are thresholded and superimposed over the MNI template brain using MRICron.

For Coh reconstructions, a robust default-mode network (15) was identified in the HC group using a seed placed in the PCC (Figure 1). Robust nodes in the network (*p* < 0.005) included not only the PCC but homologous regions of lateral parietal cortex along the border with the angular gyrus, and in the frontal lobe, a region of anterior pre-frontal cortex (aPFC) along the midline in Brodmann’s Area 10 (Figure 1). When the same network was reconstructed using ICoh, similar prominent regions were identified along medial parietal, lateral parietal, and pre-frontal cortex (Figure 1). In contrast to the Coh functional connectivity reconstruction for the DMN, very little resting-state functional connectivity was identified for voxels neighboring the PCC seed using ICoh, although separate clusters neighboring the seed were still identifiable (Figure 1). In order to estimate the degree of spatial overlap between the DMN reconstructions using Coh and ICoh, each functional connectivity map was thresholded (inclusive mask of the 5% of voxels with highest magnitude for either Coh or ICoh) and entered into a spatial conjunction analysis (Figure 1). Strong spatial overlap was seen between the Coh and ICoh connectivity maps (Figure 1, in yellow). Regions unique to the Coh DMN reconstructions (Figure 1, in red) included voxels in and around the PCC seed (pink dot, Figure 1) as well as medial and lateral parietal regions (including Brodmann’s Area 7). Using ICoh, only a few clusters of resting-state functional connectivity were identified that were unique to this metric including visual cortex and the lateral IPL (Figure 1, in green).

### Sensorimotor network

**Figure 2:**
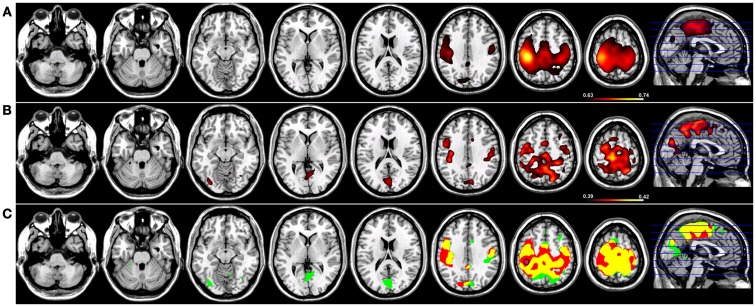
**Resting-state functional connectivity maps for a seed placed in left primary motor cortex (M1; pink circle) in 15 healthy control subjects reconstructing the sensorimotor network (SMN) for left M1**. **(A)** SMN reconstruction using magnitude coherence (Coh). **(B)** SMN reconstruction using imaginary coherence (ICoh). **(C)** Spatial conjunction of Coh maps (in red) and ICoh maps (in green) for the SMN and overlap between the two maps (in yellow). Conventions as in previous figures.

**Figure 3:**
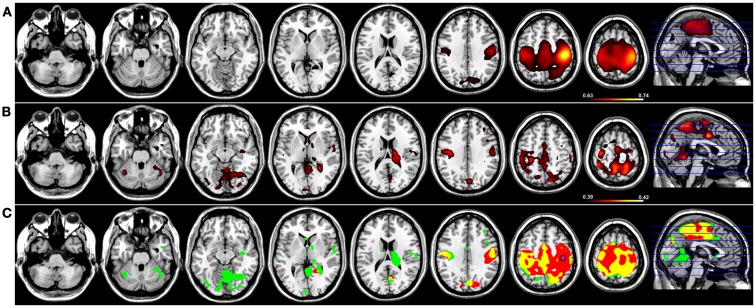
**Resting-state functional connectivity maps for a seed placed in right primary motor cortex (M1; pink circle) in 15 healthy control subjects reconstructing the sensorimotor network (SMN) for right M1**. **(A)** SMN reconstruction using magnitude coherence (Coh). **(B)** SMN reconstruction using imaginary coherence (ICoh). **(C)** Spatial conjunction of Coh maps (in red) and ICoh maps (in green) for the SMN and overlap between the two maps (in yellow). Conventions as in previous figures.

Robust SMNs were reconstructed in both groups (Figure 2) using Coh from a seed placed over the hand knob in either the left (Figure 2) or right (Figure 3) central sulcus. For a seed placed in left M1 (Figure 2) or right M1 (Figure 3) using Coh, a robust (*p* < 0.005) network was identified between the seed and M1 in the hemisphere contralateral to the seed, as well as medial parietal and frontal regions that correspond to the SMA. Like the PCC seed, the functional connectivity clusters using Coh were centered (local maxima) on the seed itself, making it difficult to discern separate neighboring pre-motor and parietal fields. When the SMNs for left and right M1 are reconstructed using ICoh, robust functional connectivity is seen between M1 and pre-motor and posterior parietal cortex bilaterally as well as SMA with little to no functional connectivity is observed between M1 bilaterally for either the left or right seed (Figures 2 and 3) making these pre-motor and parietal fields distinct. These fields included the superior parietal lobe (SPL) and middle frontal gyrus (MFG) bilaterally, the pre-SMA, midline parietal structures, ventral pre-motor (PMv) cortex, and the cerebellum. When functional connectivity maps for HC participants are thresholded and then run through a spatial conjunction analysis, extensive overlap is seen between the Coh and ICoh reconstructions of the SMN. Overlap included regions of the MFG, anterior parietal fields (e.g., Brodmann’s Areas 3/1/2), and the SMA (Figures 2 and 3, in yellow) with clusters unique to ICoh in the occipital lobes (Figures 2–3, in green) and cerebellum (Figure 3, in green). Regions unique to Coh were restricted to M1 bilaterally as surrounding fields (Figures 2 and 3, in red).

### Basal ganglia network

**Figure 4:**
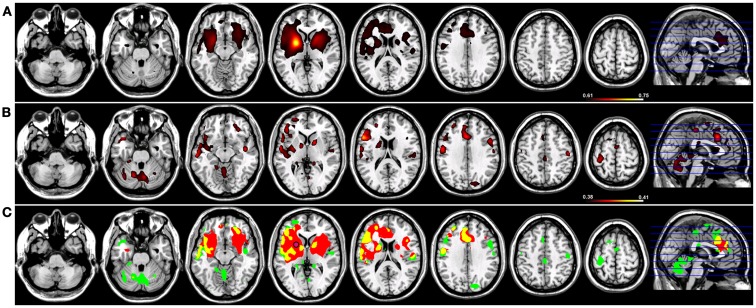
**Resting-state functional connectivity maps for a seed placed in the left putamen (pink circle) in 15 healthy control subjects reconstructing the left basal ganglia network (BGN)**. **(A)** BGN reconstruction using magnitude coherence (Coh). **(B)** BGN reconstruction using imaginary coherence (ICoh). **(C)** Spatial conjunction of Coh maps (in red) and ICoh maps (in green) for the BGN and overlap between the two maps (in yellow). Conventions as in previous figures.

**Figure 5:**
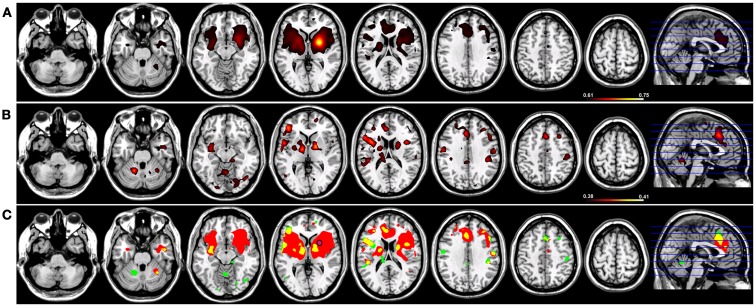
**Resting-state functional connectivity maps for a seed placed in the right putamen (pink circle) in 15 healthy control subjects reconstructing the right basal ganglia network (BGN)**. **(A)** BGN reconstruction using magnitude coherence (Coh). **(B)** BGN reconstruction using imaginary coherence (ICoh). **(C)** Spatial conjunction of Coh maps (in red) and ICoh maps (in green) for the BGN and overlap between the two maps (in yellow). Conventions as in previous figures.

When a functional connectivity seed was placed in either the left or right putamen, robust traditional resting-state functional connectivity networks were identified encompassing regions within the basal ganglia bilaterally in both groups (Figures 4 and 5). For a seed placed in either the left (Figure 4) or right (Figure 5) putamen, robust (*p* < 0.005) regions of resting-state functional connectivity included the caudate and putamen bilaterally and a region of medial pre-frontal cortex (Figure 4). Substantial seed blur was observed over the putamen seed and neighboring regions when using Coh, making the identification of neighboring functionally connected structures difficult. This blur was minimized using ICoh, where robust functional connectivity was seen between both the left (Figure 4) and right (Figure 5) seed with the bilateral caudate, cerebellum, and pre-motor cortices and midline pre-SMA. Significant overlap was seen when functional connectivity maps for Coh and ICoh were thresholded and entered into a spatial conjunction analysis for the medial pre-frontal cluster (mPFC) as well as structures in the basal ganglia (Figures 4 and 5, in yellow). Regions unique to Coh were generally restricted to regions around the basal ganglia seed (Figures 4 and 5, in red) Regions unique to ICoh included clusters in PMv and dorsal pre-motor (PMd) cortex and the cerebellum (Figures 4 and 5, in green).

### Group comparison: HC vs. tspFHD

Existing models of neuronal interaction in patients with tspFHD are centered on the working hypotheses that aberrant activity within the sensorimotor system contributes to faulty coupling between regions of the brain that are specific for hand control (1). Therefore, we predict that in patients with tspFHD, aberrant patterns of resting-state functional connectivity will be profound for primary motor cortex (SMN) and the putamen (BGN) and less pronounced for resting-state functional connectivity networks that do not directly play a role in motor control (DMN). Furthermore, based on the distinctive and restricted patterns of resting-state functional connectivity that we observe using ICoh, we also predict that specific differences in Coh will be observed between the two groups using this metric.

#### Sensorimotor network

**Figure 6:**
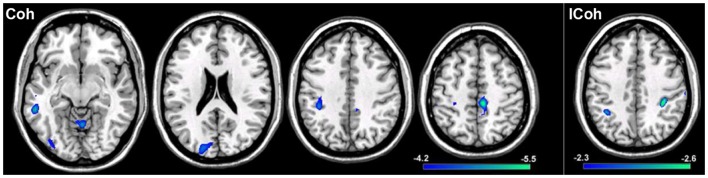
**Results from a group comparison between 11 patients with tspFHD and matched healthy controls for resting-state functional connectivity maps in the hemisphere representing the affected hand (left M1)**. Reductions in resting-state connectivity in the tspFHD group are shown in blue. Left panel: difference maps for Coh reconstructions of connections in affected M1 (left to right, *Z* = 63, 94, 112, 142). Right panel: difference maps for ICoh reconstructions of connections in affected M1 (*Z* = 115). Conventions as in previous figures.

In order to test the hypotheses that deviations in resting-state connectivity are functionally relevant to impairments in motor behavior in tspFHD, we performed a group comparison between resting-state functional connectivity maps generated from seeds placed in M1 of the hemisphere contralateral to the most affected hand (e.g., left M1) hemisphere against LM1 seed maps in a cohort of 11 HCs (see Materials and Methods). For functional connectivity maps generated using Coh, significant (*p* < 0.001) reductions in resting-state functional connectivity in the tspFHD group were observed over five regions (Figure 6, left panel). A region of the left (contralateral to the affected hand) cerebellar hemisphere (anterior lobe, culmen) was significantly underconnected in tspFHD. In the left hemisphere (contralateral to the affected hand), regions of the middle temporal gyrus (BA21), inferior parietal lobe (BA40), and the cuneus (BA18) were also significantly underconnected in the patient cohort. Finally, a region of the paracentral lobule over Brodmann’s Area 6 of the frontal lobe corresponding to the SMA along the midline was also significantly underconnected in patients with tspFHD. No regions were found to be overconnected in the group comparison of the Coh maps. When ICoh maps for the affected M1 seed are compared between the two groups, three cortical areas are significantly (*p* < 0.005) underconnected in the patient group. In the frontal lobe, a region of the MFG (BA6) at the midline, a region of the inferior parietal lobe (BA40) of the left hemisphere (contralateral to the affected hand), and a region of the right post-central gyrus (ipsilateral to the affected hand) were all underconnected with left M1 in patients with tspFHD (Figure 6, right panel). No regions were found to be significantly overconnected in the ICoh maps for the affected hemisphere in patients with tspFHD.

**Figure 7:**
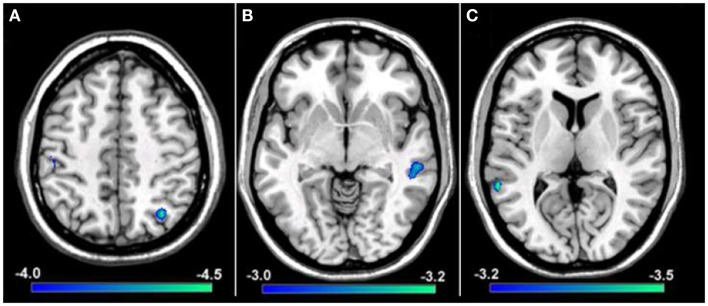
**Results from a group comparison between 11 patients with tspFHD and matched healthy controls for resting-state functional connectivity maps using Coh**. **(A)** Difference maps for Coh reconstructions of connections of the SMN representing the unaffected hand (right M1; *Z* = 120). **(B)** Difference maps for Coh reconstructions of connections of the putamen representing the affected hand (left; *Z* = 67). **(C)** Difference maps for Coh reconstructions of the default-mode network (*Z* = 63). Conventions as in Figure 6.

To explore if reductions in resting-state functional connectivity for the hemisphere contralateral to the affected hand (i.e., left hemisphere) extended to cortical representations contralateral to the unaffected hand (i.e., right hemisphere), a group comparison was performed between tspFHD patients and HCs for functional connectivity maps of right M1. Group differences were only statistically significant for the cluster in the right IPL (BA7; Figure 7A) ipsilateral to the representation of the unaffected hand, with reduced functional connectivity in the tspFHD group (*p* < 0.0025). No regions were significantly overconnected with the unaffected representation in the tspFHD group. For the group comparison of the ICoh maps of the seed placed in the hemisphere contralateral to the unaffected hand, no significant group differences were identified, even at a relaxed statistical threshold (*p* < 0.01).

#### Basal ganglia network

In order to explore if the differences in resting-state functional connectivity we observe for cortical regions of the sensorimotor system extend to deficits in coupling for regions of the basal ganglia, group comparisons were performed between Coh and ICoh maps for seeds placed in the putamen of the affected (i.e., left; contralateral to the affected hand) hemisphere. At a stringent statistical threshold (*p* < 0.001), no significant differences in resting-state functional connectivity were observed between the two groups for Coh maps of the putamen in the affected hemisphere, although at a more relaxed threshold (*p* < 0.0075) a single region was found to be significantly underconnected, in the middle temporal gyrus of the right hemisphere (Figure 7B). For the group comparison of the ICoh maps of connectivity with the putamen of the affected hemisphere, no significant differences were identified even at a relaxed threshold of *p* < 0.01. For both Coh and ICoh maps of the connectivity of the putamen in the hemisphere contralateral to the less affected hand (right hemisphere), no significant group differences were identified, even at a relaxed statistical threshold (*p* < 0.01). Therefore, in contrast to robust differences in functional connectivity identified for primary motor cortex, weak changes in functional connectivity are present within the BGN in tspFHD.

#### Default-mode network

We predict that, in contrast to robust changes in resting-state functional connectivity identified for both the affected and unaffected representations of the hand in SMN, minimal changes in coupling will be present in patients with tspFHD for a functionally irrelevant network (DMN). When a group comparison was made between the Coh maps for the DMN, no significant differences were observed using a rigorous statistical threshold (*p* < 0.001). At a more relaxed statistical threshold (*p* < 0.0075) a single cluster was significantly different, with lower Coh values in the tspFHD group, in the left superior temporal gyrus (BA22, Figure 7C). No regions were identified as being overconnected with PCC in the patient group even at a relaxed statistical threshold, and no significant differences in connectivity were observed between the two groups for a comparison of ICoh DMN maps (*p* > 0.01).

## Discussion

There are two main findings in the present study. First, using magnitude Coh and ICoh, we are able to identify robust interactions within the DMN, SMN, and BGN in both healthy individuals and individuals with tspFHD. While the spatial distribution of these networks did overlap to some degree, unique interactions not observed using Coh are identifiable in the ICoh-reconstructed networks. We also illustrate the utility of ICoh as a technique to overcome seed blur regularly observed in bivariate metrics of functional connectivity. Second, we report impoverished resting-state BOLD connectivity in tspFHD when compared to HCs across functionally relevant networks (SMN, BGN). The deficits in resting-state connectivity we observe here in patients with tspFHD are the most robust brain regions that represent the affected hand, specifically within cortical fields that serve the SMN.

### Imaginary coherence networks in fMRI

As a metric for functional connectivity, ICoh has been developed from and applied to datasets collected with high temporal resolution to correct for artifacts marked by a zero time lag, specifically in EEG (22) and MEG (27, 30, 31, 33). Here, we are able to illustrate that the same metric can be used to correct for spurious artifacts in BOLD timeseries data, such as those generated from spatial blur incorporating the seed voxel. Similar to reports using ICoh on EEG/MEG data, ICoh values can be quite low, as the imaginary portion of the Coh spectrum occupies a small (but significant) portion of the full spectra. As a result, the spatial distribution of resting-state functional connectivity maps derived from ICoh substantially overlap those generated using traditional Coh metrics. However, as the artifacts generated by the seed voxel(s) are quite strong, correcting for these artifacts permits the visualization of connectivity networks that may be otherwise sub-threshold in traditional Coh analyses. For example, ICoh reconstructions for a seed placed in PCC revealed separate clusters of connectivity outside of the traditional DMN in the anterior parietal lobe and occipital lobe bilaterally (Figure 1). Furthermore, by eliminating confounds induced by the PCC seed through ICoh, distinct clusters along the medial parietal-occipital lobes became identifiable over the anterior cerebellum and pre-cuneus, whereas these local maxima were not as readily identifiable in the Coh reconstruction. Conversely, lateral parietal regions of the DMN that show traditional coupling with the PCC (15, 24) were absent in ICoh reconstructions. Unfortunately, due to the low magnitude of ICoh values, it is also possible for this metric to miss certain structures within these networks if there is not enough power in the dataset.

Correcting for seed blur artifact is especially relevant when networks incorporate the connections between neighboring cortical fields, as is the case of both the SMN and BGN. Tracer injection studies in non-human primates have yielded a wealth of information about the connections between M1 and neighboring cortical fields (34, 35) as well as between the putamen and regions of the basal ganglia and cortex (36, 37). At the sub-cortical level, M1 is densely interconnected in a topographic fashion with both the basal ganglia and the cerebellum (38). Cortically, M1 is bidirectionally connected with regions of pre-motor cortex, midline frontal structures such as the pre-SMA and SMA, as well as higher-order somatosensory fields including BA2, BA5 (in the SPL), and BA7 (in the inferior parietal lobe). Although our resting-state reconstruction maps of M1 connectivity using Coh are dominated by interactions between M1 with the SMA and motor cortex of the contralateral hemisphere, a more distinct pattern of connections is observed for both LM1 and RM1 using ICoh that includes pre-motor and posterior parietal cortical fields (Figures 2 and 3). When seed blur is corrected in the ICoh maps, functional connections between the putamen and the ipsilateral globus pallidus are observable. In the macaque monkey, robust projections exist between the putamen and the internal and external segments of the globus pallidus (39, 40). Unique interactions within the SMN are also identified using ICoh, including connections between M1 and the pre-SMA bilaterally, fitting the known connections of the pre-SMA in the macaque monkey (41). Similarly for the BGN, ICoh maps for both the left and right putamen are distinct from Coh maps derived using the same seeds, with denser connections between the putamen and pre-frontal cortex. Projections from the putamen to pre-frontal cortical fields are consistent with the known anatomical connectivity of this structure (42). It is possible that the temporal dynamics of interactions between these structures (e.g., putamen and PFC, pre-SMA, and M1) are more accurately captured using ICoh.

The temporal scaling of the neuronal interactions that we model using ICoh are within the same magnitude as those evaluated using resting-state BOLD fMRI, i.e., in the 0.01- to 0.1-Hz range. Therefore, the timing of these interactions occurs at a very long time scale (i.e., on the order of seconds). While these interactions are robust, it is not entirely clear what these very slow interactions represent at the neurophysiological level. This is particularly challenging when evaluating resting-state data, where neural coupling is not tied to a specific behavior (43). Nonetheless, this issue is not unique to the usage of ICoh as a functional connectivity metric, and instead is ubiquitous to BOLD functional imaging designs in general. Future studies will need to evaluate the functional significance of oscillatory activity in the brain at this very slow time scale.

### Changes in resting-state functional connectivity specific to tspFHD

When either Coh or ICoh is applied to resting-state BOLD data, both groups (HC, tspFHD) show robust sensorimotor, basal ganglia, and DMNs with no apparent reorganization of these systems. Significant differences between these networks emerge with respect to the magnitude of activity within each system, with robust reductions in functional coupling in the tspFHD cohort. The greatest reduction in resting-state functional connectivity between the two groups was observed for the connections of M1 representing the most affected hand (Figure 6). Where group differences for the Coh reconstructions were widespread, reductions in resting-state functional connectivity identified using ICoh were more specific, and limited to interactions between motor cortex, anterior parietal fields and medial PFC.

Reductions in resting-state functional connectivity are likely related to aberrant (increased) patterns of cortical activity identified during motor behavior in patients with tspFHD. Increased activation in M1 has been identified using functional neuroimaging techniques such as fMRI and PET (5, 44). Hyperactivity in the cortical motor system has also been reported in tspFHD using transcranial magnetic stimulation (TMS). TMS studies have demonstrated that individuals with tspFHD are deficient in their ability to suppress motor activity following activation [intra-cortical inhibition; (45)]. Data from TMS studies have also demonstrated that these patients have insufficient center-surround inhibitory mechanisms typically present in motor cortex (46) and compromised sensorimotor integration in the cortex (47). TMS studies pairing magnetic stimulation with median nerve stimulation have provided evidence for maladaptive plasticity in motor cortex in tspFHD, specifically with a lack of spatial specificity in sensorimotor cortex (48). Increased gain in the sensorimotor system induced by aberrant activity and neuroplasticity in motor cortex may lead to inefficient coupling between brain regions that comprise this network, further leading to the reductions in resting-state functional connectivity we report here. Interestingly, group differences using ICoh were only significant for regions of the cortex representing the most affected hand, suggesting a functional substrate for the clinical deficits reported in these patients.

Between-group differences for the putamen seed contralateral to the most affected hand were only statistically significant using Coh. It is possible that, since ICoh occupies a small portion of the Coh spectrum (as mentioned above), greater power is needed in our dataset in order to detect changes using ICoh. It is also possible that group differences for the connections of the basal ganglia are only captured using Coh, and that time-lagged interactions captured through ICoh remain uncompromised in task-specific dystonia. Alternatively, although increased neural firing has been reported in the basal ganglia in tspFHD (9, 44, 49) it is possible that hyperactivity in the basal ganglia does not directly translate into resting-state deficits in functional connectivity in either metric. This may be due to the pronounced reorganization in motor and somatosensory cortex present in patients with tspFHD, a result of aberrant neuroplasticity within the cortical motor system (11, 50, 51).

### Clinical implications

Our main clinical finding, that reductions in resting-state functional connectivity in ICoh were robust for motor cortical fields representing the most affected hand (and relatively preserved for brain regions serving control of the less affected hand) suggest a possible functional substrate for motor deficits in tspFHD. However, the exact functional significance of this relationship remains unclear – as our sample size was limited and heterogeneous it was not possible to relate specific symptoms to changes in functional connectivity. Future studies will need to correlate the magnitude of functional connectivity deficits to specific clinical impairments found in this disorder.

Resting-state functional connectivity has the potential to act as a reliable measure of monitoring neural plasticity during training, as it is not dependent on subject compliance or task design (17). Many recent studies are beginning to demonstrate that changes in resting-state functional connectivity are related to improvements in both motor (52, 53) and cognitive (54, 55) function following training. Therefore, one possible clinical application for resting-state functional imaging in movement disorders like tspFHD is tracking neuroplasticity during recovery. Sensory and motor training paradigms clinically designed to restore function in dystonia effectively attempt to “re-map” aberrant topography within the cortical fields contralateral to the affected digits (56, 57). It is possible that, in addition to reorganizing the topography of the cortex and “quiescing” hyperactivity in the brain, these sensorimotor learning behavioral interventions could remediate coupling we see reduced through ICoh between higher-order parietal fields and the motor cortices representing the affected hand. For example, these regions in anterior parietal cortex with insufficient coupling in dystonia are known to control parameters of hand control (35), incorporating kinesthetic feedback, and cognitive parameters such as spatial attention (58, 59). Therefore, sensorimotor learning paradigms should focus on modulating inputs outside of simple sensory and motor discrimination, possibly through training prehension of the hand and multisensory attention.

### Limitations

Here, we provide a case for ICoh as a functional connectivity metric to be used to correct for artifacts in BOLD timeseries data induced by spurious correlations in the BOLD signal (including seed blur). Furthermore, the differences in resting-state functional connectivity between groups we identify using ICoh are more precise and interpretable than those using Coh, speaking to the power of this metric to detect disease-specific changes. However, there are some considerations when dealing with the imaginary component of the Coh spectrum. The magnitude of ICoh values are known to be lower and less normally distributed than more traditional measures of functional connectivity, including Coh (30). Data transformation and non-parametric statistical estimation can correct for some of these limitations. Furthermore, the functional significance of time-lag in BOLD timeseries data, where the acquisition is on the order of 1–3 s, is not entirely clear. It is possible that these temporally dependent interactions derived using ICoh are not the result of direct, monosynaptic connections between brain structures and instead represents downstream stages of processing.

It is also possible that the demographics of the patient cohort may also play a role in discovering differences between brain networks. In unique movement disorders like tspFHD, it becomes difficult to recruit a homogenous sample for a single site due to differences in illness severity, duration, and the characteristics of the disorder (e.g., musician’s cramp vs. writer’s cramp). However, the ability to identify robust changes in functional connectivity, even in this small sample, speak to the profound reductions in neural interactions underlying a loss of motor control in tspFHD.

## Conclusion

We report that in tspFHD, the resting-state functional connectivity between brain regions representing the affected hand are compromised. However, future studies will need to relate the underconnectivity to specific sensory and motor impairments in tspFHD. We also show that a novel functional connectivity metric, ICoh, can be used to examine complementary interactions that are not always observed in full-spectrum Coh.

## Conflict of Interest Statement

The authors declare that the research was conducted in the absence of any commercial or financial relationships that could be construed as a potential conflict of interest.
